# Spinsterhood and its impact on disease features in women with rheumatoid arthritis

**DOI:** 10.1186/1477-7525-9-58

**Published:** 2011-08-01

**Authors:** Yousra Ibn Yacoub, Bouchra Amine, Assia Laatiris, Najia Hajjaj-Hassouni

**Affiliations:** 1 Department of Rheumatology (Pr N. Hajjaj-Hassouni), El Ayachi Hospital, University Hospital of Rabat-Sale, Morocco

## Abstract

**Objective:**

To evaluate the impact of spinsterhood on disease characteristics and quality of life (QoL) in Moroccan women with rheumatoid arthritis (RA).

**Methods:**

185 women with RA were recruited in this study. Marital status was specified as follow: 1. Spinsterhood (never-married woman aged 38 or over), 2. Distressed marriage; 3. Nondistressed marriage and 4. Divorced or widowed. Marital distress was assessed by a self-report concerning coping efficacy and burden caring of husbands. Assessment criteria included the evaluation of: age at onset (years), diagnosis delay (months), disease duration (years), disease activity (evaluated by physical examination, biological inflammatory tests; and disease activity score (DAS 28)), pain intensity (measured with a visual analogue scale (VAS)); and radiographic damage (evaluated by the Sharp's method as modified by van der Heijde). Treatments (doses and duration) were specified. The Health Assessment Questionnaire (HAQ) was used to evaluate functional disability. QoL was measured using the Arabic version of the generic instrument SF-36.

**Results:**

In our data, spinsterhood was detected in 42 (22.7%) patients vs. 88 (47.5%) with distressed marriage, 28 (15.1%) with nondistressed marriage and 27 (14.6%) divorced or widowed. Comparing the 4 groups, we found that QoL in never-married women was damaged in a significant way comparing to the other groups. Mental as well as physical aspects were affected. Also, we found that spinsterhood was associated to an early age at onset (p = 0.009), pain intensity (p < 0.001); clinical (p < 0.001) and biological disease activity (C-reactive protein; p = 0.02) and functional disability (p < 0.001). Logistic regression analysis revealed a significant relationship between spinsterhood and early age at onset and severe functional disability (for all p ≤ 0.01).

**Conclusion:**

This study suggests that spinsterhood in our RA patients was associated with an altered QoL even compared with distressed married women. Also, we state that spinsterhood was associated with an early age at onset, severe joint pain; higher disease activity and with altered functional ability. It seems important to consider not only disease-related parameters but also social status as a determinant factor of poor course in RA.

## Introduction

Rheumatoid arthritis (RA) is a chronic inflammatory disease which has great impact on general well-being, functions of daily life and the fulfillment of social roles [1]. The course of RA is multi-dimensional and varies greatly among patients [2,3]. Beside disease-related parameters, sociodemographic factors may affect disease activity, function and general well-being in RA [1,3]. It has been suggested that marital status could be associated with worse outcome in women with RA and that marriage may be associated with a lower rate of progression of functional disability in persons with RA [3,4]. However, in spite of previous data, it remains difficult to conclude whether or not marital status is a determinant of severity of RA [3].

In the Arab region, demographic patterns of marriage status have changed significantly in recent decades [5]. Early marriage has declined sharply in parts of the Arab world and the rate of celibacy or spinsterhood has increased significantly [5]. To our knowledge, no Maghrebean or Arabic data have focused on the impact of spinsterhood on disease characteristics among women with RA. The aim of the present study was to evaluate spinsterhood and its relationships with disease variables and quality of life (QoL) in Moroccan women with RA.

## Patients and methods

### Patients

185 women with the diagnosis of RA were recruited consecutively at the Department of Rheumatology of El Ayachi Hospital in the University hospital of Rabat-Sale in Morocco between October 2009 and November 2010. Informed consent was obtained from all patients and the local medical Ethics Committee approved the study. Patients were recruited in consultation or during hospitalization. For all patients were collected: age, educational level (subdivided into three groups: no formal education, primary education; and secondary education or more), socioeconomic status (determined by the monthly household income (less than 1000 Dirhams (DH), between 1000 and 2000 DH; 2000 and 5000 DH and above 5000), disease duration (years), diagnosis delay (months), morning stiffness (minutes), night pain (number of awakenings) and pain intensity (visual analogue scale (VAS): 0-100 mm, 0 = no pain and 100 = severe pain imaginable). Disease activity was evaluated clinically by the duration of morning stiffness (minutes), night pain, number of swollen and tender joints; biologically by the erythrocyte sedimentation rate (ESR) and C reactive protein (CRP); and by the disease activity score (DAS 28). For structural damage, radiographs of hands, wrists and feet were evaluated by one observer and scored using Sharp's method as modified by van der Heijde [6]. Functional disability was assessed by using the Moroccan version of Health Assessment Questionnaire (HAQ) [7].

Were collected also: extra-articular manifestations, immunological abnormalities (rheumatoid factor rate (RF) and anti-cyclic citrullinated protein (CCP) antibody positivity by Elisa method), and treatment (doses and duration) with corticosteroids, disease modifying anti rheumatic drugs (DMARD's) and biologic agents. The quality of life (QoL) was evaluated using the Arabic version of the Medical Outcomes Study Short Form 36 Health Survey: the SF-36; validated in Morocco [8].

### Marital status

Marital status was categorized in four groups: 1. Spinsterhood (never married woman aged 38 or over), 2. Distressed marriage; 3. Nondistressed marriage and 4. Divorced or widowed. Never-married women younger than 38 years-old were excluded from the sample. Marital distress was assessed by a self-report concerning coping efficacy and burden caring of husbands.

#### Statistical analysis

The statistical analyses were carried out using the SPSS 13 for Windows (SPSS Inc., Chicago, IL, USA). Data for patients were presented as mean and standard deviations for continuous variables and as frequencies and percentages for categorical variables. Differences within the 4 groups were assessed by using the ANOVAs Analysis of variance. Logistic regression was used to assess disease parameters related to spinsterhood. Results of logistic regression were presented as odds Ratios (OR) with 95% confidence intervals (CI). Odds Ratios were adjusted for the potential confounders (age, educational level, socioeconomic status and treatment) to examine the independent association between spinsterhood and disease parameters. The significance level of P was set at 0.05.

## Results

In our sample, the mean age of patients was 46.57 ± 10.78 years. The mean age at onset was 35.3 ± 12 years and the mean disease duration was 10.58 ± 8.13 years. Spinsterhood was detected in 42 (22.7%) patients vs. 88 (47.5%) with distressed marriage, 28 (15.1%) with nondistressed marriage and 27 (14.6%) divorced or widowed. In ANOVAs Analysis of variance performed with Bonferroni correction, we found that spinsterhood was associated to an early age at onset (p = 0.009), pain intensity (VAS pain) (p < 0.001); clinical and biological disease activity (DAS28 (p < 0.001) and CRP (p = 0.02)) and functional disability (HAQ) (p < 0.001). Patients' characteristics and disease-related variables according to the marital status are summarized in table 1. There were no statistically significant differences in structural damage, immunological status, extra-articular manifestations or treatment modalities between the four groups.

**Table 1:** Study population characteristics according to the marital status (n = 185)

Characteristics	Spinsterhood	Distressed marriage	Nondistressed marriage	Divorced or widowed	P
**Educational level**					
-No formal education	15 (35.71%)	39 (44.31%)	19 (67.85%)	22 (81.48%)	0.010
-Primary education	11 (26.19%)	26 (29.54%)	2 (7.14%)	2 (7.4%)	
- ≥ Secondary education	16 (38.1%)	23 (26.13)	7 (25%)	3 (11.11%)	
**Household income (DH)**					
≤ less than 1000	20 (47.62%)	44 (50%)	11 (39.28%)	12 (44.45%)	0.042
> 1000- < 2000	15 (35.72%)	30 (34.1%)	7 (25%)	11 (40.75%)	
≥ 2000- < 5000	5 (11.9%)	9 (10.22%)	4 (14.28%)	3 (11.1%)	
≥ 5000	2 (4.76%)	5 (5.68%)	6 (21.42%)	1 (3.7%)	
**Treatment**					
-Corticosteroids	40 (95.23%)	82 (93.18%)	24 (85.71%)	26 (96.29%)	0.139
-DMARD's	36 (85.71%)	78 (88.63%)	23 (82.14%)	25 (92.59%)	0.282
-Biologic agents	7 (16.66%)	12 (13.63%)	4 (14.28%)	1 (3.7%)	0.096
**Age (years)**	44.33 ± 2.54	39.21 ± 6.48	47.27 ± 3.22	55.76 ± 5.66	0.003
**Age at onset (years)**	29.81 ± 11.65	36.52 ± 11.76	34.6 ± 12.23	34.72 ± 12.34	0.009
**VAS pain intensity (0-100)**	67.14 ± 18.11	55.14 ± 16.08	53.63 ± 12.01	52.37 ± 14.21	< 0.001
**Morning stiffness(min)**	72.05 ± 16.38	47.16 ± 14.66	48.37 ± 11.25	45.79 ± 16.54	0.013
**Night pain (Number)**	2.08 ± 0.47	1.42 ± 0.76	1.54 ± 0.57	1.44 ± 0.48	0.006
**Tender joints (0-28)**	14.02 ± 4.47	11.45 ± 3.27	12.07 ± 3.7	12.2 ± 3.65	0.021
**Swollen joints (0-28)**	7.97 ± 5.25	5.89 ± 2.42	5.3 ± 2.27	5.47 ± 3.1	0.004
**ESR (mm)**	47.30 ± 17.44	47.71 ± 18.37	48.79 ± 16.31	45.85 ± 19.17	0.062
**CRP (mg/l)**	37.07 ± 19.17	29.74 ± 13.26	30.09 ± 10.95	28.61 ± 11.93	0.02
**Rheumatoid factor (UI/L)**	80.52 ± 45.75	88.19 ± 39.55	88.02 ± 43.13	89.75 ± 40.07	0.203
**AntiCCp antibodies (UI/L)**	127.46 ± 104.36	160.2 ± 93.02	131.57 ± 97.53	110.97 ± 107.66	0.081
**DAS 28**	6.32 ± 1.5	5.17 ± 1.3	4.97 ± 1.94	5.04 ± 1.26	< 0.001
**HAQ (0-3)**	2.09 ± 0.58	1.39 ± 0.56	1.36 ± 0.68	1.23 ± 0.87	< 0.001
**Sharp total score**	98.45 ± 39.87	91.45 ± 35.39	91.34 ± 34.60	89.58 ± 38.61	0.317

DH, Dirhams; VAS, visual analogue scale; ESR, erythrocyte sedimentation rate; CRP, C-reactive protein; DAS, disease activity score; HAQ, health assessment questionnaire.

P ≤ 0.05: significant

Also, spinsterhood was associated to lower scores of QoL comparing to the other groups (Table 2). Mental as well as physical aspects were affected in a significant way in never-married women. The logistic regression analysis revealed a significant independent relationship between spinsterhood and early age at onset [OR = 1.217-CI (95%) 1.136-1.303] and severe functional disability (HAQ) [OR = 2.772-CI (95%) 1.217-1.669] (for all p ≤ 0.01). Likewise, in regression analysis, spinsterhood was associated with altered scores of the domain of mental health [OR = 1.086-CI (95%) 1.033-1.413] and social functioning [OR = 2.953-CI (95%) 0.710-0.998] of SF-36 (for all p ≤ 0.001).

**Table 2:** Scores of quality of life (SF-36) according to the marital status

Domains of SF-36	Spinsterhood	Distressed marriage	Nondistressed marriage	Divorced or widowed	P
**Physical functioning**	39.88 ± 25.86	55.31 ± 23.83	57.55 ± 27.58	59.49 ± 28.47	< 0.001
**Role limitation**	36.30 ± 27.53	63.51 ± 22.05	66.11 ± 23.39	65.72 ± 29.02	0.001
**Role emotional**	38.46 ± 27.65	72.85 ± 26.67	73.32 ± 24.52	76.9 ± 24.77	0.003
**vitality**	32.28 ± 27.09	43.45 ± 17.6	44.46 ± 19.48	45.67 ± 20.08	< 0.001
**Mental health**	41.26 ± 10.55	54.51 ± 10.04	58.39 ± 10.54	57.25 ± 12.31	< 0.001
**Social functioning**	44.53 ± 17.5	65.42 ± 17.28	67.9 ± 18.94	68.74 ± 15.17	< 0.001
**Bodily pain**	35.70 ± 20.33	64.13 ± 20.1	66.14 ± 18.24	65.14 ± 16.46	0.002
**General health**	41.77 ± 15.46	64.57 ± 17.42	67.1 ± 20.49	66.17 ± 18.36	< 0.001

P ≤ 0.05: significant

## Discussion

In our sample, we state the high prevalence of spinsterhood among Moroccan women with RA comparing to the general population (approximately 16%) [5]. Spinsterhood seems to be associated with joint pain, high disease activity and worse functional disability in our RA patients. In their study, Reese et al suggested that relationships may influence adjustment to chronic pain conditions such as RA [9]. Also, marital status and marital adjustment have been considered to be related to higher pain, higher disease activity and severe physical and psychological disability in previous data [3,4,9,10]. Moreover, as in our study, other authors have found that unmarried women had poorer HAQ scores [4,11]. However, it has been shown that being married in itself is not associated with better health in RA but that being in a well-adjusted or nondistressed marriage is linked with less pain and better functioning [11]. In fact, the associations between spinsterhood and disease progression and severity in RA are complex and interpretation requires simultaneous assessment of a variety of other variables [10].

In our data, never married women had worse disease features even compared with distressed married women. Cultural, social, behavioral and religious characteristics and particularities in Arab societies may explain our results [12].

Also, the negative impact of spinsterhood on patients' QoL has been reported previously in RA patients [1,13]. Limitations in basic function in RA patients can undermine well-being and contribute to poor QoL, perpetuating limitations in important social roles and functions of daily life and increasing disability that, in turn may engender poor mental and physical health [1,13].

Our results suggest that spinsterhood seems to be a determinant factor of poor course in our RA. Those findings may underscore the importance of considering not only disease parameters but also social status particularly in our context and may inform clinical interventions of RA. Large studies seem to be necessary in order to confirm those results.

## Competing interests

The authors declare that they have no competing interests.

## Authors' contributions

BA and NHH conceived the study, participated in its design and have been involved in drafting the manuscript and revising it critically for intellectual content. YIY and AL did data management, statistical analysis and interpretation of results. YIY wrote the manuscript with the collaboration of all authors. All authors have given final approval of the manuscript.
